# Release–Chelation Effect of Ultrasound-Assisted Citric Acid Treatment on Heavy Metal Removal Efficiency in *Pyropia haitanensis*

**DOI:** 10.3390/toxics14050401

**Published:** 2026-05-07

**Authors:** Wenhui Cui, Sibi Huang, Huaqing Zeng, Yutong Li, Leyi Zheng, Changhua Xu

**Affiliations:** 1 College of Food Science & Technology, Shanghai Ocean University, Shanghai 201306, China; 2 National R&D Branch Center for Freshwater Aquatic Products Processing Technology (Shanghai), Shanghai 201306, China; 3 Qinpu Biotechnology Pte Ltd., Suzhou 215200, China; 4 Quanzhou Fugangjia Food Co., Ltd., Jinjiang, Quanzhou 362000, China

**Keywords:** *Pyropia haitanensis*, UACA, heavy metal removal, process optimization

## Abstract

*Pyropia haitanensis* (*P. haitanensis*) is a commercially significant alga in China, and the vitality of its industry is closely associated with coastal economic stability and food safety. Intensifying coastal pollution and deteriorating aquaculture conditions have exacerbated heavy metal accumulation in *P. haitanensis*, threatening sustainable development and limiting industry viability. Conventional processing methods exhibit substantial limitations in heavy metal removal, nutrient retention, and preservation of algal tissue structure, restricting their ability to meet market demands. To address this challenge, we developed a synergistic ultrasound-assisted citric acid treatment technology. In this process, ultrasound facilitates the release of heavy metal from algal tissue, while citric acid chelates and removes them, termed the “Release–Chelation” effect. The approach aims to efficiently remove heavy metal while maintaining *P. haitanensis* quality and nutritional value. Under optimized conditions of citric acid concentration, sonication time, and power, heavy metal residues were effectively reduced below national regulatory standards. SEM and FT-IR analyses indicate that removal occurs with minimal structural damage. Nutritional analyses revealed slight reductions in crude protein and amino acid content, yet overall nutritional quality remained satisfactory. These results demonstrate that the technology preserves the edible value of *P. haitanensis* while efficiently removing heavy metals, highlighting its potential to advance the algae industry toward safer and more sustainable practices.

## 1. Introduction

*Pyropia haitanensis* (*P. haitanensis*), a commercially significant large-scale alga in Asia, has been cultivated in East and Southeast Asia for millennia and represents a highly valuable marine crop [1]. In China, cultivation of *P. haitanensis* is mainly concentrated in the coastal regions of Fujian, Zhejiang, and Guangdong provinces, providing a major source of income for local fishermen. Recently, its cultivation has gradually expanded to northern regions. *P. haitanensis* is rich in bioactive compounds, including proteins, polysaccharides, polyphenols, and vitamins, which confer high nutritional value [2]. Moreover, the proteins in *P. haitanensis* provide a complete spectrum of amino acids in substantial quantities, and the alga exhibits bioactivities including anti-cancer, antioxidant, hypoglycemic, and immunomodulatory effects [3]. It fulfills the demand for high-quality food and is widely favored by consumers.

In recent years, the rapid development of industry and agriculture has led to increasingly significant environmental issues, such as the burning of fossil fuels, mining, and the use of pesticides and chemical fertilizers. In addition, improper treatment of industrial and agricultural wastewater has caused heavy metals to accumulate in lake basins [4]. Heavy metals deposited in soil and the atmosphere are leached into aquatic ecosystems through rainwater erosion, which may lead to heavy metal contamination of surface and groundwater in rivers, lakes, and oceans [5,6]. Due to the structure of its cell walls, which readily adsorb heavy metals from seawater, algae are considered biological indicators of heavy metals in seawater [7]. The cell wall of *P. haitanensis* consists of multiple layers of microfibers, including cellulose, pectin, and algal polysaccharides [8,9], forming a porous network structure with a large surface area and high viscosity. Due to the presence of various functional groups in the cell wall, such as carbonyl, hydroxyl, carboxyl, amino, and phosphate groups, *P. haitanensis* exhibits strong adsorption capacity for heavy metal ions [10]. This characteristic makes *P. haitanensis* prone to accumulating harmful metals such as lead, cadmium, and arsenic, thereby posing food safety risks. Long-term exposure to heavy metals can cause damage to the nervous system and liver function, respiratory diseases, and carries a risk of inducing cancer [11,12]. In recent years, as the consumption of *P. haitanensis* has increased and given that heavy metals are difficult to biodegrade, they have come to threaten the entire marine ecosystem and human health through the food chain [13]. As one of the algal species with significant economic value in Asia [14], reducing heavy metal residues in *P. haitanensis* is currently one of the most pressing issues to be addressed.

Ultrasonication, as an emerging cleaning technology, can extend food shelf life while effectively removing sediment and impurities. Moreover, ultrasonic heating facilitates the release of heavy metal ions from food, thereby reducing their residual levels [15]. Additionally, specific food additives, including citric acid (CA) [16] and acetic acid (HAc) [17], are applied during the cleaning process to remove sediment and concurrently reduce heavy metal content. These reagents primarily immobilize heavy metals via chelation through -COOH and -OH functional groups or by inducing precipitation. Consequently, ultrasonic and chemical cleaning technologies exhibit considerable potential for the removal of heavy metals from food. Amir et al. [18] reported that CA could reduce residual levels of Hg, Pb, Zn, and As in spinach by 7–54%. Porova et al. [19] applied ultrasonic technology to decontaminate milk from heavy metals. This approach effectively reduced the levels of Pb, As, Hg, and other heavy metals in milk, while significantly altering the particle size distribution of fat and protein, yielding a more uniform emulsion. Although these techniques effectively reduce heavy metal levels in food, their application in algae remains limited. Currently, strategies for removing heavy metals from algae primarily involve ultrasonic treatment, alone or combined with mild heating and/or ethylenediaminetetraacetic acid (EDTA), which can reduce As, Cd, I, and Hg levels in kelp [20]. However, these methods can adversely affect quality attributes, including morphology, nutritional composition, and color.

Building on previous research, this study developed an ultrasound-assisted citric acid (UACA) synergistic treatment system. The system primarily employs the cavitation effect of ultrasound and the shear forces generated by microjets to disrupt the binding of heavy metal ions with polysaccharides, proteins, and other cellular components in *P. haitanensis*, thereby facilitating their elution. CA, as a tricarboxylic acid chelating agent, can form stable complexes with heavy metal ions released into the solution. Furthermore, H^+^ ions produced by CA dissociation compete with heavy metal ions for binding sites via protonation [21], thereby occupying some sites and further reducing the availability of binding sites for metal ions in the algal matrix, ultimately lowering residual heavy metal contents in *P. haitanensis*. In summary, the mechanism of heavy metal removal is referred to as the “Release–Chelation” effect. Implementation of this system enables efficient removal of heavy metals from *P. haitanensis,* providing a feasible and environmentally friendly approach to support its sustainable and safe cultivation.

## 2. Materials and Methods

### 2.1. Sample Preparation

The *P. haitanensis* samples used in this study were collected from the first harvest of 2024 at a farm located in the waters off Xiapu, Fujian. After collection, the fresh samples were washed, air dried, sealed in food grade bags, and stored at −20 °C. Prior to analysis, samples collected from different areas of the farm were thoroughly mixed to ensure homogeneity and subsequently thawed at room temperature before experimentation.

### 2.2. Determination of Heavy Metal Content

Following the method described by Karizza F. Catenza et al. [22] with minor modifications, a three-step digestion procedure was employed to process the *P. haitanensis* samples. Microwave digestion was performed using a 1:8 ratio of *P. haitanensis* samples to nitric acid. After cooling, the digest was transferred to an evaporating dish and concentrated to approximately 1 mL at 140 °C until no solid precipitate remained. The volume was then adjusted to 25 mL with deionized water, mixed thoroughly, and a blank control was included.

The concentration of a specific element in a sample was calculated using the following formula:

(1) $$X=\frac{(\rho -\rho_{0})\times V\times f}{m\times 1000}$$

where *X* is the concentration of the element of interest in the sample, mg/kg; *ρ* is the mass concentration of the element of interest in the sample solution, µg/L; *ρ*_0_ is the mass concentration of the element of interest in the sample blank solution, µg/L; *V* is the final volume of the sample digestion solution, mL; *f* is the dilution factor of the sample; *m* is the mass of the sample, g; 1000 is the conversion factor.

The formula for calculating the heavy metal removal rate in the sample is as follows:

(2) $$Removal\;rate\left(\%\right)=\frac{X_{1}-X_{0}}{X_{1}}\times 100\%$$

where *X*_1_ is the element content in the sample before treatment, mg/kg; *X*_0_ is the element content in the sample after heavy metal removal, mg/kg.

### 2.3. Reagents Used

All reagents used in this study were of analytical grade. The heavy metal mixed standard solution was obtained from Shanghai Titan Technology Co., Ltd. (Shanghai, China). CaCl_2_, EDTA, lactic acid (LA), sucrose, copper sulfate, potassium sulfate, HAc, concentrated sulfuric acid, and hydrochloric acid were purchased from Sinopharm Chemical Reagent Co., Ltd. (Shanghai, China). H_2_O_2_ was obtained from Guanjia Chemical Reagent (Chenzhou, China). Tris-HCl was purchased from Yuanye Biotechnology Co., Ltd. (Shanghai, China). Glutaraldehyde, anhydrous ethanol, tert-butanol, and phenol were supplied by Shanghai McLean Biochemical Technology Co., Ltd. (Shanghai, China).

### 2.4. Chemical Removal Agents Used for the Removal of Heavy Metals

Following the method of Amir [18] with slight modifications, aqueous suspensions of *P. haitanensis* were prepared at a solid to liquid ratio of 1:50 (*w*/*v*). CA, EDTA, H_2_O_2_, HAc, and LA were used as removal agents, and the suspensions were treated for 30 min to remove Cr, As, Cd, and Pb from *P. haitanensis*.

### 2.5. Physical Removal Methods

#### 2.5.1. Ultrasound

Following the method described by Condón Abanto [23] with slight modifications, aqueous suspensions of *P. haitanensis* were prepared at a solid to liquid ratio of 1:50 (*w*/*v*). Ultrasonic treatments were performed at power levels of 100, 150, 200, 250, and 300 W for 20, 30, and 40 min to evaluate the removal of Cr, As, Cd, and Pb. The corresponding removal rates were calculated using Formula (2).

#### 2.5.2. Mechanical Stirring

Following the method described by Lan [24] with slight modifications, aqueous suspensions of *P. haitanensis* were prepared at a solid to liquid ratio of 1:50 (*w*/*v*). Treatments were conducted at rotation speeds of 100, 200, 300, 400, and 500 rpm for 20, 30, and 40 min to assess the removal of Cr, As, Cd, and Pb. The corresponding removal rates were calculated using Formula (2).

### 2.6. Single-Factor Experiments

Because physical removal methods or chemical reagents alone could not reduce residual heavy metal levels in *P. haitanensis* below the national standard limits, a single-factor experiment was conducted to evaluate the combined use of UACA. Based on the preceding results, CA concentration, ultrasonic power, and sonication time were selected as the experimental factors at levels of 0.02, 0.06, 0.10, 0.14, and 0.18 mol/L; 180, 210, 240, 270, and 300 W; and 10, 15, 20, 25, and 30 min, respectively. The solid-to-liquid ratio was fixed at 1:50 (*w*/*v*), and the removal rates of Cr, As, Cd, and Pb were used as evaluation indices.

### 2.7. Response Surface Design

Based on the results of the single-factor experiment, a three-factor, three-level experiment was conducted using CA concentration, sonication time, and ultrasound power (denoted as factors A, B, and C, respectively) as experimental factors (Table 1). The removal rates of Cr, As, Cd, and Pb in the samples were used as performance indicators.

### 2.8. Optimization Process Validation Experiment

The removal process was optimized using Design Expert 12 software by analyzing the interactions among the three factors. The effectiveness and accuracy of the optimized model were subsequently validated through three parallel experiments.

### 2.9. Fourier Transform Infrared Spectroscopy (FT-IR)

Following the method described by Younis [25] with minor modifications, the dried sample powder was mixed with dried KBr powder at a ratio of 1:100, thoroughly ground, and pressed into disks for transmittance measurements.

### 2.10. Subcellular Fractionation

Following the method described by Zhao et al. [26], samples of *P. haitanensis* before and after treatment were weighed and homogenized in pre-chilled extraction buffer at a ratio of 1:20 (*w*/*v*). The buffer contained 250 mmol/L sucrose, 50 mmol/L Tris-HCl (pH 7.5), and 1 mmol/L dithiothreitol. After centrifugation at 1500 rpm for 30 s, the resulting pellet was collected as the cell wall fraction. The supernatant was further centrifuged at 12,250 rpm for 45 min, and the final supernatant was collected as the cytoplasmic soluble fraction.

### 2.11. Scanning Electron Microscopy (SEM)

Following the method described by Nguyen [27] with minor modifications, the samples were fixed overnight in 2.5% glutaraldehyde at 4 °C. The samples were then dehydrated sequentially in 30%, 50%, 70%, 80%, and 90% ethanol solutions for 15 min each, followed by two washes with anhydrous ethanol for 20 min each. Subsequently, the samples were transferred to mixtures of anhydrous ethanol and tert-butanol at ratios of 3:1, 1:1, and 1:3 for 15 min each, followed by immersion in pure tert-butanol for 20 min. The processed samples were freeze-dried for at least 48 h.

### 2.12. Quality Changes

Protein, polysaccharides, and amino acid contents were determined according to the methods described by Qi [28] and Vanessa [29], with minor modifications. The nutritional quality of amino acids was evaluated according to the amino acid scoring model recommended by FAO/WHO, and the amino acid score (AAS) of *P. haitanensis* was calculated using the following formula:

$$AAS=\frac{Content\;of\;a\;specific\;amino\;acid\;in\;the\;protein\;of\;P.\;haitanensis}{Content\;of\;the\;corresponding\;amino\;acid\;in\;the\;reference\;protein}\times 100$$

### 2.13. Statistical Analysis

All experiments were performed in triplicate. Experimental results were expressed as the mean ± standard deviation. Statistical analyses were performed using SPSS 23.0 and GraphPad Prism 9.50. Differences among groups were evaluated by one-way analysis of variance, and *p* < 0.05 was considered statistically significant. Microsoft Excel 2022 and GraphPad Prism 9.50 were used to generate the relevant figures.

## 3. Results and Discussion

### 3.1. Comparison of Chemical Removal Efficacy Agents

**Figure 1:**
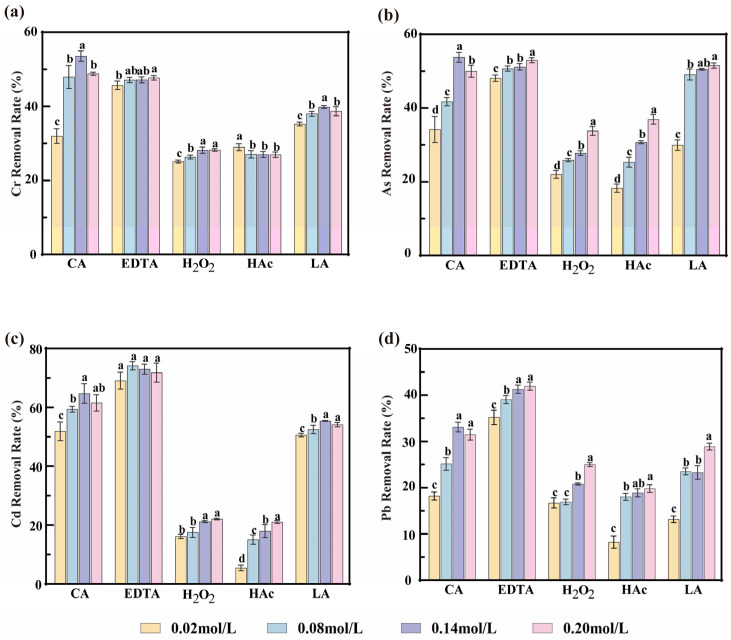
Effects of different chelating removal agents on the removal efficiency of heavy metals from *P. haitanensis*. (**a**–**d**) illustrate the comparative removal efficiencies of Cr, As, Cd, and Pb, respectively, from *P. haitanensis* using different chelating agents. Different letters indicate significant differences in removal efficiency among treatments with different concentrations of the same chelating removal agents (*p* < 0.05).

To systematically evaluate the effects of different agents on heavy metal removal from *P. haitanensis*, five commonly used chemical removal agents, namely CA, EDTA, H_2_O_2_, HAc, and LA, were selected. The removal efficiencies of Cr, As, Cd, and Pb were compared at concentrations ranging from 0.02 to 0.20 mol/L, and the results are presented in Figure 1. Different chemical removal agents promoted the migration and release of heavy metals from *P. haitanensis* to varying extents, resulting in significant differences in removal efficiency among treatments. Similar findings were reported by Amir [18], who showed that chemical detergents promoted the transfer of Hg, Pb, As, and Zn from spinach into the liquid phase, with the effect depending on reagent type and concentration.

H_2_O_2_ and HAc showed relatively low efficiency for heavy metal removal from *P. haitanensis*, suggesting that they are unsuitable for practical application. By contrast, CA, EDTA, and LA exhibited greater removal potential, mainly through complexation via -COOH and -OH functional groups, displacement reactions, and metal precipitation [30]. Among these agents, EDTA showed the highest removal capacity; however, the sample surface turned brown, and the soaking solution became green during treatment, indicating substantial color deterioration. Similar effects were reported by Kamal [31], who found that high concentrations of EDTA significantly reduced chlorophyll a and b contents in plants, resulting in tissue discoloration and fading. Therefore, although EDTA is highly effective in removing heavy metals, it markedly impairs the visual quality of the product.

In contrast, LA and CA are biodegradable and environmentally friendly agents that can bind metal ions to form stable complexes, thereby promoting the dissolution and transformation of heavy metals from *P. haitanensis* [32]. In addition, their relatively mild properties help preserve the original color of *P. haitanensis* to a certain extent. Figure 1 shows that although LA exhibited good removal efficiency at higher concentrations, Krook et al. [33] systematically compared the effects of LA and CA on the storage quality of the brown alga *Saccharina latissima*. Their study showed that treatment with high concentrations of LA resulted in quality deterioration during storage and imparted a fermented odor to the algal tissue. Therefore, although LA has potential for heavy metal removal, it may also cause flavor deterioration, which is consistent with the observations of the present study. As a tricarboxylic acid-based organic chelating agent, CA contains -COOH and -OH groups that can form stable chelates with heavy metal ions, thereby promoting the release of metals from the algal matrix [34,35]. As the CA concentration increased, its binding capacity for heavy metal ions also increased, thereby facilitating complex formation. At a CA concentration of 0.14 mol/L, the removal efficiencies of Cr, As, Cd, and Pb reached their optimal values, with removal rates of 53.58%, 53.79%, 64.67%, and 33.12%, respectively. This result is consistent with the trend reported by Ke [36].

In summary, among those studied, CA exhibited the highest heavy metal removal efficiency for *P. haitanensis* within the tested concentration range. At 0.14 mol/L, the residual levels of Cr, As, Cd, and Pb in *P. haitanensis* were 0.80, 1.02, 0.20, and 0.63 mg/kg, respectively. These findings indicate that treatment with CA alone, within the investigated conditions, was insufficient to reduce heavy metal levels below the limits specified by the National Standards of the People’s Republic of China, namely 0.5 mg/kg for Cr, As, and Pb, and 0.1 mg/kg for Cd.

### 3.2. Comparison of Physical Methods

#### 3.2.1. Ultrasound

**Figure 2:**
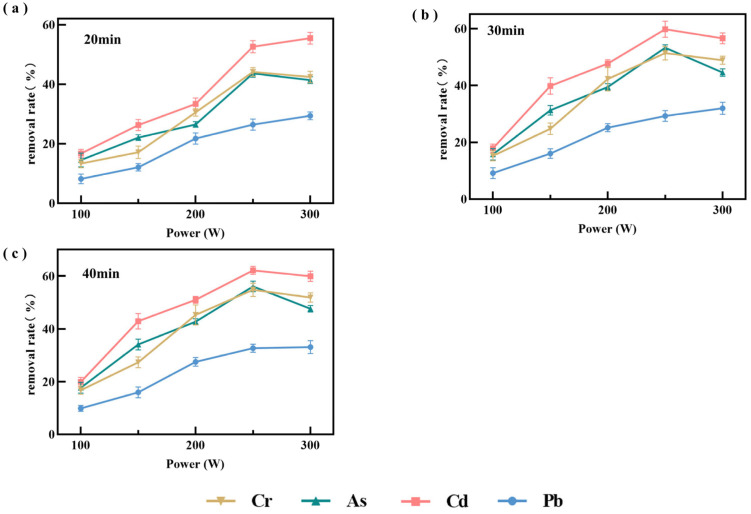
Effects of different ultrasonic power levels on the removal efficiency of heavy metals from *P. haitanensis.* (**a**–**c**) show the effects of ultrasonic power on heavy metal removal efficiency at 20, 30, and 40 min, respectively.

As a non-thermal processing technology, ultrasonication can minimize quality deterioration associated with conventional processing while preserving nutritional properties. Accordingly, the effect of ultrasonic power on the removal of heavy metals from *P. haitanensis* was systematically evaluated (Figure 2). The removal rates of the tested metals increased significantly (*p* < 0.05) as ultrasonic power increased from 100 to 250 W and then decreased slightly at higher power levels. In addition, little difference was observed between treatments of 30 and 40 min, suggesting that the system had approached equilibrium. This trend may be attributed to the high pressure and microjets generated by ultrasonic cavitation, which disrupt the cell walls and cellular structures of *P. haitanensis*, thereby exposing metal binding sites and promoting desorption and diffusion of metal ions [37]. Enhanced cavitation at moderate power levels may also intensify ion drift, collision, and frictional elution, thereby facilitating ion migration into the solution [38]. However, further increases in ultrasonic power to 250–300 W reduced removal efficiency, possibly because excessive power altered protein conformation [39,40], induced reactions in polysaccharides, and promoted cavitation bubble collapse. These changes may mask functional groups involved in metal binding or cause reabsorption of released metal ions, thereby reducing the removal rate [40].

In summary, under the tested conditions of 30 min and 250 W, the removal efficiencies of all tested heavy metals were relatively higher than those observed under the other treatment conditions. Therefore, an ultrasonic power range of 180–300 W was selected for subsequent experiments. Under these conditions, the residual concentrations of Cr, As, Cd, and Pb in *P. haitanensis* were 0.81, 1.04, 0.29, and 0.65 mg/kg, respectively, indicating that ultrasonication alone could not reduce heavy metal concentrations below the national standard limits.

#### 3.2.2. Mechanical Stirring

This study also evaluated the effectiveness of mechanical agitation, and the corresponding results are provided in the Supplementary Materials. The data indicate that the heavy metal removal efficiency achieved by mechanical agitation was significantly lower than that achieved by ultrasonic treatment. Therefore, this method is not discussed in detail in the main text.

### 3.3. UACA Single-Factor Experiment

#### 3.3.1. CA Concentrations

**Figure 3:**
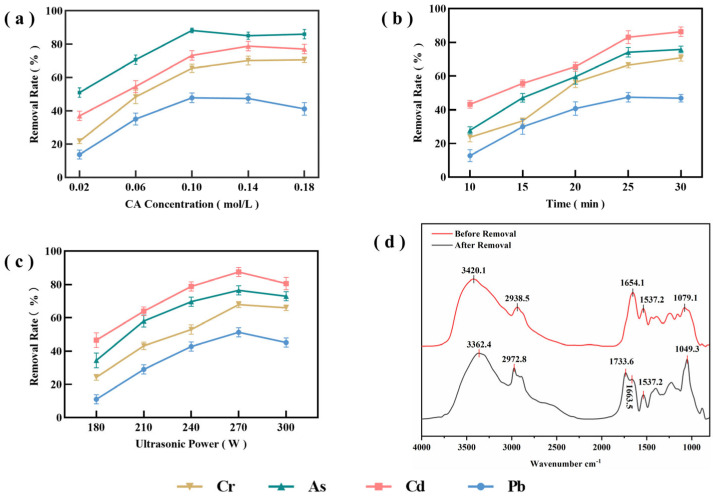
Effects of CA concentration, sonicatio time, and ultrasonic power on heavy metal removal efficiency, and FT-IR spectra of *P. haitanensis* before and after treatment. Panels (**a**−**c**) show the effects of CA concentration, sonication time, and ultrasonic power on heavy metal removal efficiency. Panel (**d**) shows the FT-IR spectra of *P. haitanensis* before and after treatment.

The removal rates of Cr, As, Cd, and Pb at different CA concentrations are presented in Figure 3a. When the CA concentration increased from 0.02 to 0.10 mol/L, the removal rates of all four heavy metals increased significantly (*p* < 0.05). This may be because, at lower CA concentrations, the limited number of CA molecules in the solution is insufficient to fully complex the metal ions, thereby restricting removal efficiency. As the CA concentration increased, -COOH and -OH groups formed stable complexes with metal ions, thereby significantly enhancing removal efficiency [41]. With further increases in CA concentration, the removal rates of Cr and Cd gradually leveled off and then declined. This may be attributed to saturation of the available binding sites on CA for metal ions, resulting in a plateau in removal rates. In contrast, the removal rates of As and Pb first decreased and then leveled off. This may be related to the relatively weaker complexation of Pb^2+^, As^3+^, and As^5+^ with the available binding sites. At excessively high CA concentrations, competitive adsorption among CA molecules may occur, and the process may be further constrained by kinetic factors such as solution viscosity [42], thereby reducing removal efficiency. It is also possible that ultrasonic cavitation weakens at high CA concentrations, thereby reducing the contact efficiency between metal ions and organic acids [43,44]. In addition, in high concentration systems, competitive adsorption and self-association among CA molecules may reduce the number of effective contact sites for metal ions in *P. haitanensis* [41].

Consequently, excessively high CA concentrations may weaken cavitation, hinder mass transfer, and reduce the synergistic removal efficiency. At 0.10 mol/L, the concentrations of Cd and Pb were 0.09 and 0.49 mg/kg, respectively, both below the national standard limits, whereas the concentrations of Cr and As were 0.59 mg/kg, which remained close to the national standard limits. Taking these factors into consideration, subsequent experiments were conducted within a CA concentration range of 0.06–0.14 mol/L for further investigation.

#### 3.3.2. Sonication Time

As shown in Figure 3b, the removal rates of the four heavy metals generally increased initially and then leveled off with increasing sonication time. Between 10 and 25 min, the removal rates of all four heavy metals increased significantly, with the largest increase observed for Cd. This suggests that ultrasonic cavitation, in combination with the chelating action of CA, can effectively promote the desorption and diffusion of metal ions from *P. haitanensis* tissue. As sonication time increased, the removal rates of most metal ions slowed and showed no further significant increase. This may be attributed to the microjets and transient high-pressure shocks generated by ultrasonic cavitation [45,46], which caused partial rupture of the cell walls of *P. haitanensis* and made internally bound heavy metals more susceptible to desorption through CA complexation. With longer sonication time, metal ions in the extracellular layer were gradually desorbed, whereas internal diffusion became increasingly limited, causing the system to approach equilibrium [47]. The removal rates of As, Cd, and Pb gradually leveled off, whereas that of Cr continued to increase slightly. This may be because complexes formed between Cr^3+^ and organic matter on the cell walls of *P. haitanensis* are relatively stable. Under UACA treatment, the desorption of Cr appears to be more strongly controlled by diffusion and coordination kinetics than that of the other metal ions, resulting in a slower and more sustained release pattern [48].

In summary, after 30 min of treatment, the concentrations of Cr and Pb in *P. haitanensis* were 0.50 and 0.49 mg/kg, respectively, both of which met the national standard limits, whereas the concentrations of As and Cd were 0.53 and 0.12 mg/kg, respectively, remaining close to the national limits. Because longer sonication time may cause greater damage to cellular structure, subsequent experiments were conducted within a sonication time range of 20–30 min.

#### 3.3.3. Ultrasound Power

As shown in Figure 3c, the removal rates of the four heavy metals generally increased initially and then decreased with increasing ultrasonic power. At ultrasonic power levels from 180 to 270 W, the removal rates of all metals increased significantly (*p* < 0.05), suggesting that enhanced cavitation within the tested range was beneficial for the removal of heavy metals. However, when ultrasonic power was further increased, the removal rates decreased slightly. Compared with treatment using CA or ultrasound alone, the UACA system produced higher removal rates for all four heavy metals. This may be attributed to ultrasonic cavitation, which disrupts some binding structures and exposes additional active sites [49], thereby enhancing complexation between CA and heavy metal ions. In addition, the energy generated by low-frequency ultrasound may cause only limited damage to *P. haitanensis* cells. Extensive rupture of cell walls, membranes, or covalent bonds may not occur under these conditions, which may help improve heavy metal removal efficiency while reducing severe loss of cellular contents [50]. At 300 W, excessive cavitation may induce a series of adverse effects, including localized heating, partial degradation of CA, and generation of cavitation-induced free radicals such as ·OH and ·H, all of which may impair the chelating capacity of CA [51]. Berrak [52] also reported that excessively high ultrasonic power could disrupt the chemical bonds formed between metal ions and adsorbent molecules, thereby hindering metal ion removal, which is consistent with the present results.

Comparison of the experimental data showed that after UACA treatment at 270 W, the concentrations of Cd and Pb in *P. haitanensis* decreased to 0.09 and 0.46 mg/kg, respectively, both below the national standard limits, whereas the concentrations of Cr and As remained close to the national limits at 0.57 and 0.52 mg/kg, respectively. Therefore, UACA treatment showed a marked effect on reducing heavy metal levels in *P. haitanensis*. Accordingly, subsequent experiments were conducted within an ultrasonic power range of 240 to 300 W.

### 3.4. Response Surface Results and Analysis

Based on the results of the single-factor experiments, the Box–Behnken design was applied to optimize the UACA process for the removal of four heavy metals.

As shown in Table 2 and Table 3, the model was highly significant (*p* < 0.001), whereas the lack of fit was not significant (*p* > 0.05), indicating that the quadratic model adequately described the experimental data. In addition, both R^2^ and adjusted R^2^ were greater than 0.8, suggesting that the model accounted for a substantial proportion of the observed variability. The Adeq Precision value was also above the critical threshold, further supporting the adequacy of the model. The normality and homoscedasticity tests of the model residuals indicate that the residuals are approximately normally distributed with no obvious outliers, demonstrating that the established quadratic regression model satisfies the underlying assumptions. The corresponding plots are included in the Supplementary Materials and can be used for further analysis and prediction. Overall, the fitted quadratic regression model was considered suitable for subsequent analysis and process optimization of heavy metal removal from *P. haitanensis*.

#### 3.4.1. Cr Removal Efficiency

**Figure 4:**
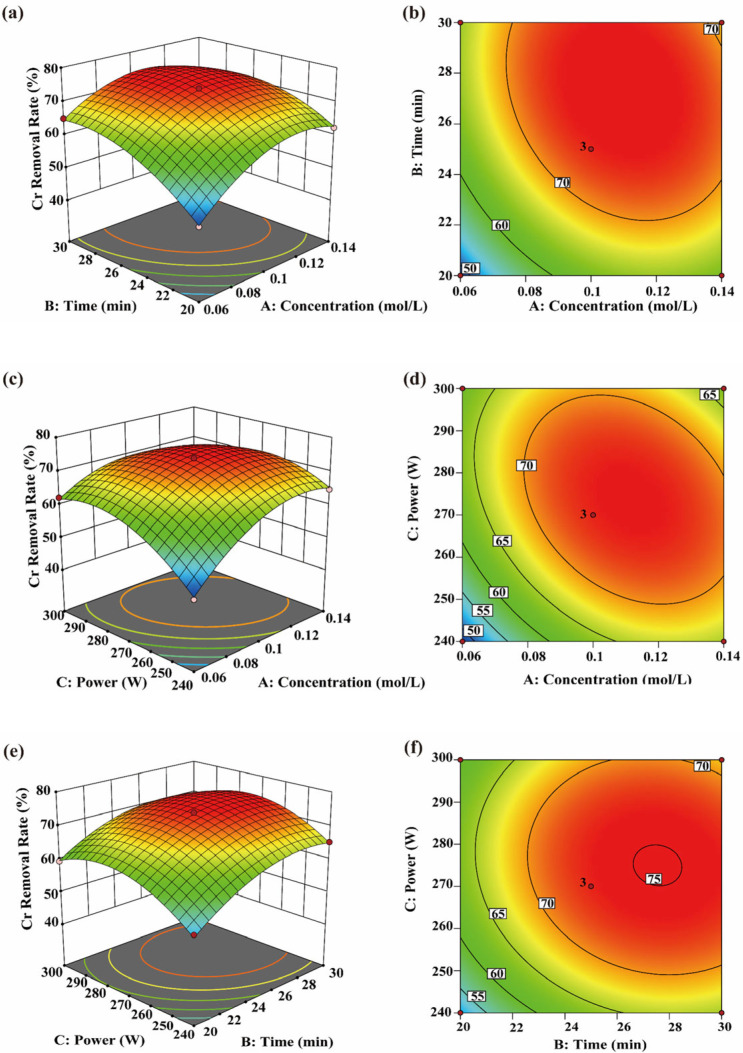
Interaction effects of CA concentration, time, and ultrasonic power on Cr removal efficiency. (**a**−**f**) show the three-dimensional response surfaces and two-dimensional contour plots of the interaction effects of AB, AC, and BC on Cr removal efficiency.

Within the investigated range of the single-factor experiments, CA concentration, sonication time and ultrasonic power significantly affected the Cr removal rate (*p* < 0.05). Specifically, the Cr removal rate increased initially and then decreased with increasing sonication time and ultrasonic power (Figure 4a–d), suggesting that the effect of ultrasonication was dependent on the tested treatment range. Moderate ultrasonication may enhance the elution efficiency of CA, thereby improving Cr removal under the tested conditions. This is consistent with the findings of Lu [34], who reported that CA can enhance Cr elution when combined with ultrasonication.

From a kinetic perspective, ultrasound may disrupt algal cell structures through the generation of microbubbles, high shear forces, and solution jets, thereby weakening the interactions between cell-wall-bound organic matter and Cr ions. This facilitates contact and complexation between CA and desorbed Cr ions, thereby promoting desorption of heavy metals from cellular tissues. Peng et al. [53] also reported that ultrasonic cavitation can disrupt cellular structures. Figure 4e,f and Table 3 show that the interaction between sonication time and ultrasonic power was not significant (*p* = 0.0553), indicating that these two factors acted relatively independently on the Cr removal process within the studied range. Ultrasonic power primarily affects the intensity of cavitation, whereas sonication time determines its cumulative extent [54]. Because these two factors operate through different mechanisms, their interaction did not reach statistical significance. However, their individual effects remained significant, and each contributed to improved Cr removal. Therefore, ultrasonic parameters should be adjusted according to practical requirements to balance Cr removal efficiency and potential adverse effects caused by excessive ultrasonic energy.

#### 3.4.2. As Removal Efficiency

**Figure 5:**
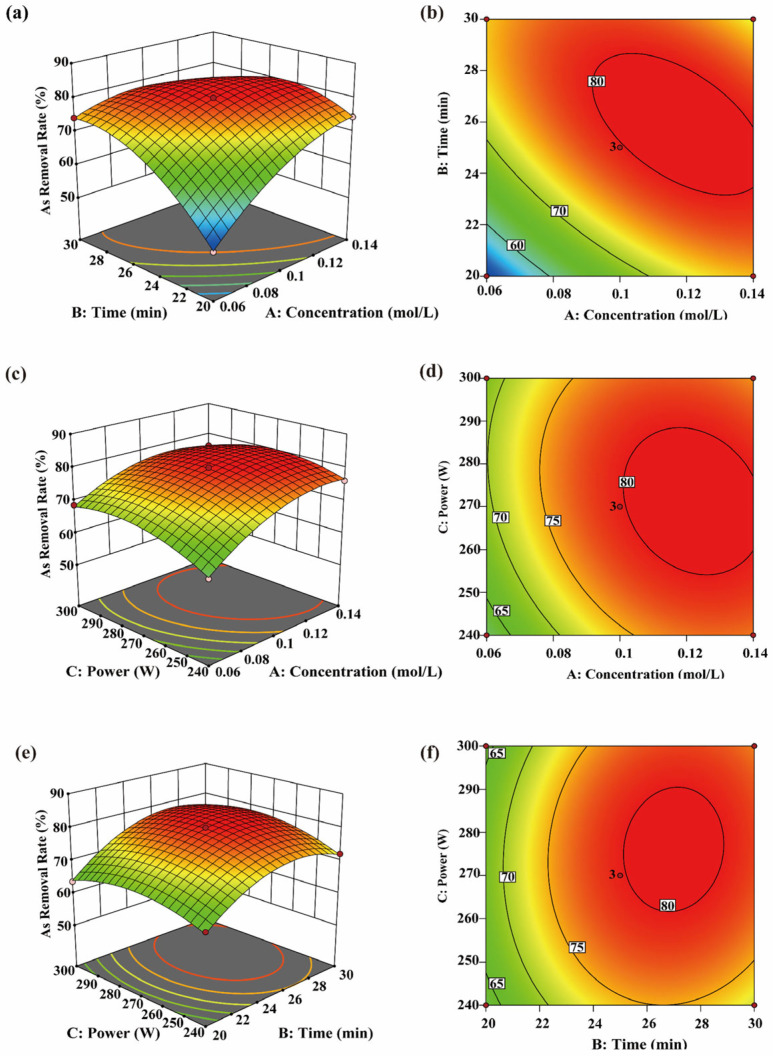
Interaction effects of CA concentration, time, and ultrasonic power on As removal efficiency. (**a**−**f**) show the three-dimensional response surfaces and two-dimensional contour plots of the interaction effects of AB, AC, and BC on As removal efficiency.

The response surface analysis shown in Figure 5 indicates that all factors significantly affected arsenic removal (*p* < 0.05), suggesting that these factors interact in influencing removal efficiency. The arsenic removal rate increased with rising CA concentration, whereas variations in sonication time and ultrasonic power initially increased and then decreased, indicating that moderate ultrasonic treatment facilitates arsenic release and migration. Arsenic removal depends partly on structural loosening and environmental changes, consistent with the general mechanism in which ultrasound disrupts cellular structures and promotes substance migration [55].

Longer sonication time allows high CA concentrations to engage more effectively in complexation reactions, promoting the transfer of arsenic from tissue into solution and reflecting the “Release–Chelation” effect of the UACA system. Estefanía [20] reported that at higher organic acid concentrations, combined treatment produced higher arsenic removal than either physical or chemical treatment alone. Similarly, Park [43] found that arsenic removal in *Hizikia fusiformis* was more effective under heat treatment. The interaction between ultrasonic power and sonication time, as shown in Figure 5e,f, significantly affected arsenic removal (*p* < 0.05). This may be due to partial conversion of acoustic energy into heat, which modifies the local environment [56] and facilitates the release of soluble arsenic and its complexation with CA. This conclusion was further supported by Wang [57]. Therefore, arsenic removal is influenced by the combined effects of CA concentration, ultrasonic power, and sonication time. Adjusting these parameters within the tested ranges can further improve removal efficiency.

#### 3.4.3. Cd Removal Efficiency

**Figure 6:**
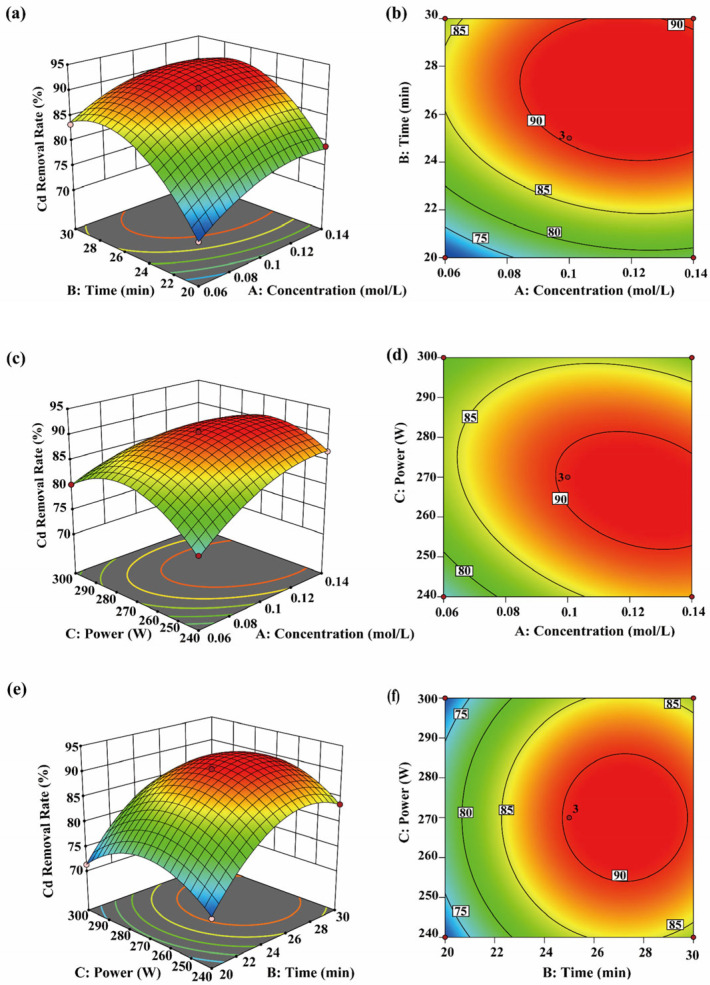
Interaction effects of CA concentration, time, and ultrasonic power on Cd removal efficiency. (**a**−**f**) show the three-dimensional response surfaces and two-dimensional contour plots of the interaction effects of AB, AC, and BC on Cd removal efficiency.

The results in Figure 6a–d show that the Cd removal rate gradually increases and then stabilizes with increasing sonication time, suggesting that a dynamic equilibrium between removal and complexation is established in the system. Longer sonication time promotes sustained cavitation within the algal cells, facilitating gradual release of bound Cd and enhancing its migration into solution [20]. This observation is consistent with the findings of S. Condón-Abanto [23]. CA enhances chelation of various heavy metals and increases Cd solubility by modulating system acidity and promoting complexation [58]. Under lower acidity, H^+^ and Cd^2+^ compete for adsorption sites on cell wall proteins and polysaccharides. This competition weakens the bond between Cd and the matrix, facilitating chelation by CA and migration of Cd into solution [59]. Analysis of Figure 6e,f and Table 3 indicates that the interaction between sonication time and ultrasonic power was not significant (*p* = 0.9703 < 0.05), suggesting that both factors mainly influenced Cd removal through their individual effects. This finding is consistent with observations in the Cr system. Therefore, during process optimization, sonication time and ultrasonic power can be adjusted independently to balance removal efficiency and energy consumption.

#### 3.4.4. Pb Removal Efficiency

**Figure 7:**
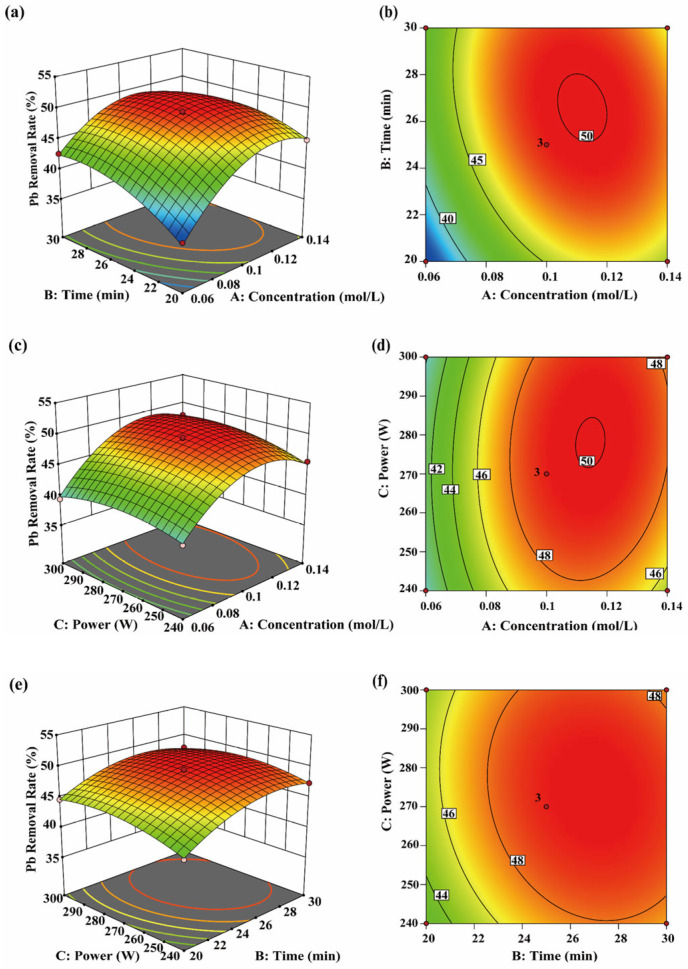
Interaction effects of CA concentration, time, and ultrasonic power on Pb removal efficiency. (**a**−**f**) show the three-dimensional response surfaces and two-dimensional contour plots of the interaction effects of AB, AC, and BC on Pb removal efficiency.

As shown in Figure 7a,b, CA concentration and sonication time significantly affected Pb removal (*p* < 0.05). The interaction between CA concentration and sonication time initially enhanced Pb removal; however, further increases in these parameters led to stabilization of the removal rate, suggesting a dynamic equilibrium for Pb in the system. Under low pH and the cumulative effect of cavitation, Pb^2+^ mobility increases, promoting the formation of stable complexes with CA and reducing residual Pb in *P. haitanensis* cells [60]. With longer sonication time, the interaction between CA concentration and exposure duration contributed to further Pb desorption, enhancing removal efficiency. This effect is likely related to cavitation, including the frequency and intensity of microjets and shock waves, which weaken interactions between Pb and its binding sites, promoting desorption [20]. While ultrasonic power partially influences cell structure disruption [61], the interaction between CA concentration and ultrasonic power did not significantly affect Pb removal. Main effects analysis indicates that sonication time (B) has a stronger influence than ultrasonic power (C), suggesting that cumulative exposure time is more critical for Pb desorption and migration in this system. Therefore, during process optimization, ensuring an appropriate sonication time is a priority to fully utilize cavitation-enhanced effects, while ultrasonic power should be adjusted to avoid potential adverse effects from excessive sonication.

#### 3.4.5. Optimization Process Validation Study

Using Design Expert, the optimized conditions for UACA treatment were determined as a CA concentration of 0.108 mol/L, a sonication time of 27.88 min, and an ultrasonic power of 269.37 W. Under these conditions, the removal rates were 75.3% for Cr, 80.7% for As, 92.1% for Cd, and 50.0% for Pb. Considering laboratory constraints and practical feasibility, the removal conditions were adjusted to a CA concentration of 0.11 mol/L, a sonication time of 27 min, and an ultrasonic power of 270 W. Under these adjusted conditions, the removal rates for Cr, As, Cd, and Pb were 74.8%, 81.2%, 92.0%, and 50.5%, respectively. The residual concentrations of Cr, As, Cd, and Pb were 0.43 mg/kg, 0.42 mg/kg, 0.058 mg/kg, and 0.47 mg/kg, respectively, all below national standard limits and in general agreement with predicted values. These results indicate that the optimized parameters are reliable and demonstrate practical applicability.

### 3.5. FT-IR Analysis

Figure 3d shows the infrared spectra of *P. haitanensis* before and after UACA treatment. The results indicate that the combined UACA system removes heavy metals primarily by altering the structure of major functional groups in *P. haitanensis*. The -OH stretching peak shifted from 3420.1 cm^−1^ to 3362.4 cm^−1^, suggesting that hydrogen bonding interactions may have been rearranged, and that the intermolecular or intramolecular hydrogen bonding environment was altered. The Amide I band shifted from 1654.1 cm^−1^ to 1663.5 cm^−1^, indicating that the secondary structure of proteins may have undergone conformational changes. A new absorption peak appeared at 1733.6 cm^−1^, close to the characteristic ester -C=O peak, which may be associated with interactions between the carboxyl group of CA and hydroxyl groups of polysaccharides or proteins, suggesting possible partial esterification [62]. The absorption peak at 1079 cm^−1^, corresponding to nucleic acids, phosphoproteins, and phosphoglycans, disappeared, while a new -C-O-H vibrational peak appeared at 1049 cm^−1^. This indicates that some phosphorus-containing functional groups may have been disrupted, and that polysaccharide chains may have experienced local breaks or conformational changes, resulting in loosening of the cell wall structure and facilitating complex formation between CA and metal ions [63]. The -C-H absorption peak at 2972 cm^−1^ shifted to 2938 cm^−1^, suggesting alterations in the vibrational environment of the -C-H bond, which may reflect loosening of lipid or hydrophobic segment arrangements. These observations support the conclusion that the cell wall and membrane structures underwent relaxation and rearrangement. Overall, UACA treatment appears to enhance heavy metal removal efficiency by modifying the characteristics of functional groups, loosening the cell wall structure, and altering the microenvironment of heavy metal binding sites.

### 3.6. Subcellular Distribution of Heavy Metals

**Figure 8:**
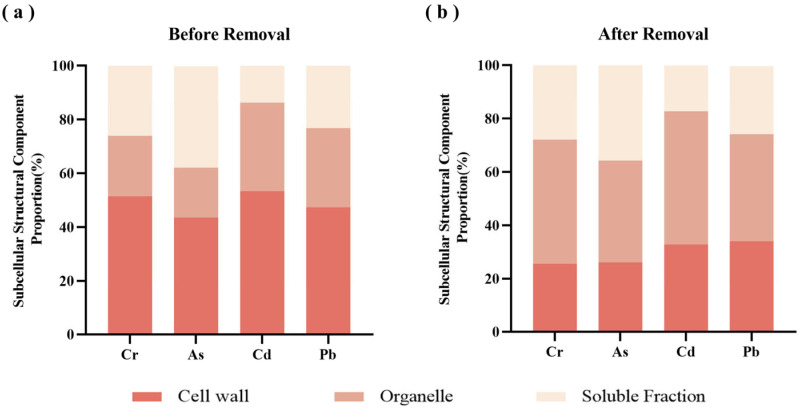
Subcellular distribution of heavy metals in *P. haitanensis* before and after UACA treatment. (**a**,**b**) show the distribution of heavy metals among different subcellular components of *P. haitanensis* before and after processing, respectively.

This study analyzed the subcellular distribution of four heavy metals, Cr, As, Cd, and Pb, in *P. haitanensis* before and after UACA treatment (Table 4 and Figure 8). Before treatment, Cr, As, Cd, and Pb were mainly located in the cell wall and organelles, with the cell wall containing the largest proportion. Specifically, Cr and Pb comprised over 50% of the total, consistent with Zhao [26]. This indicates that, before treatment, heavy metals were mainly bound to the cell wall through functional groups such as polysaccharides and proteins.

Following UACA treatment, heavy metal concentrations in all subcellular fractions of *P. haitanensis* decreased significantly, while their relative distribution proportions changed notably. Specifically, the proportion of heavy metals in organelles increased, followed by that in the cell wall fraction. This change did not reflect an actual increase in metals in organelles but resulted from preferential removal from the cell wall, leading to a relative increase in the proportion of remaining heavy metals in organelles [64]. Within organelles, heavy metals predominantly exist in stable chelated forms, such as complexes with phytochelatins, glutathione, and metal transport-related proteins [65], making them less susceptible to removal by the UACA system. Consequently, during the overall removal process, heavy metals in organelles exhibit relatively lower removal efficiency, leading to an increase in their relative proportion. Overall, UACA treatment demonstrates selectivity in heavy metal removal across subcellular components, preferentially targeting weakly bound, readily exchangeable metals in the cell wall, while removal of stably bound metals in organelles is limited. Therefore, subsequent experiments can adjust treatment conditions to address this limitation and achieve more thorough heavy metal removal.

### 3.7. SEM Analysis

**Figure 9:**
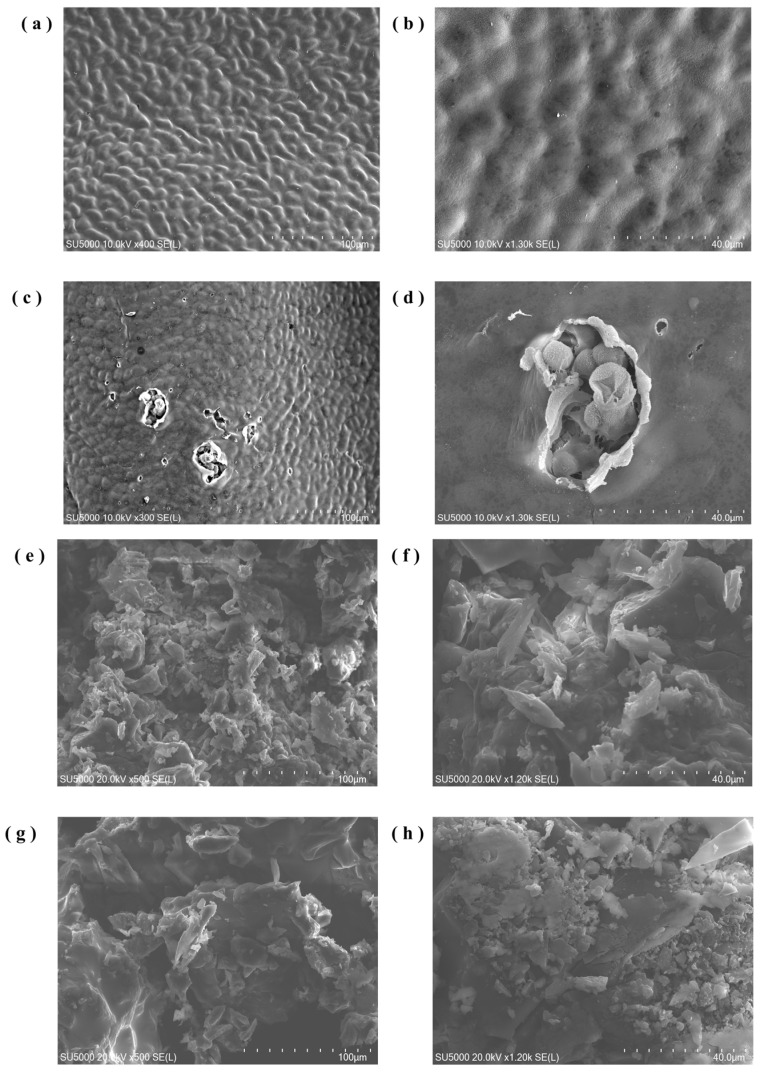
SEM comparison of surface structures of *P. haitanensis* before and after UACA treatment. (**a**–**h**) show SEM images of untreated fresh *P. haitanensis*, UACA-treated *P. haitanensis*, CA solution, and heavy metal-containing washing solutions, respectively, at different magnifications.

This study investigated changes in the surface structure of *P. haitanensis* under UACA treatment using SEM. The results indicate that dehydration significantly altered the microstructure of the alga surface. Figure 9a,b shows SEM images of fresh *P. haitanensis*, exhibiting a relatively smooth and uniform surface, indicating intact cell walls. Under higher magnification, the surface morphology displayed regular, smooth folds with no apparent damage. Figure 9c,d show localized fractures and cracks on the surface of *P. haitanensis* after UACA treatment, indicating that the treatment disrupted cell wall integrity in specific regions. At higher magnifications, cell wall and membrane structures were observed to be severely damaged. These localized alterations likely increased cell wall porosity. These observations are consistent with FT-IR results, which indicated localized breaks or exposure of polysaccharide chains and protein structures, resulting in a looser cell wall structure. These structural changes facilitate CA complexation with metal ions, thereby enhancing heavy metal removal.

CA occurs naturally in fruits and vegetables; however, the CA used as a food additive is primarily synthetic [66]. Accordingly, the composition of CA and the *P. haitanensis* lavage solution was also examined (Figure 9e–h). The analysis indicated that during washing of *P. haitanensis* with CA, most CA remained in the washing solution, with negligible residual CA on the algal surface. These results suggest that CA effectively removes heavy metals from *P. haitanensis* without leaving residual CA, thereby minimizing potential health risks associated with CA residues.

### 3.8. Quality Changes

The data in Table 5 indicate that, following heavy metals removal, the overall contents of crude protein and polysaccharides in *P. haitanensis* decreased, with reductions of 13.67% and 14.34%, respectively. Table 6 shows that essential amino acids (EAA), non-essential amino acids (NEAA), and total amino acids (TAA) all decreased after treatment, with retention rates remaining above 78%. According to the FAO/WHO ideal model, high-quality protein contains approximately 40% essential amino acids relative to total amino acids, with an essential-to-non-essential amino acid ratio exceeding 60%. The results indicate that in *P. haitanensis*, after heavy metals removal, the EAA/NEAA and EAA/TAA ratios remained relatively stable, with the EAA/NEAA ratio exceeding 60% and the EAA/TAA ratio around 40%, suggesting that the protein retained high quality and met FAO/WHO standards.

**Figure 10:**
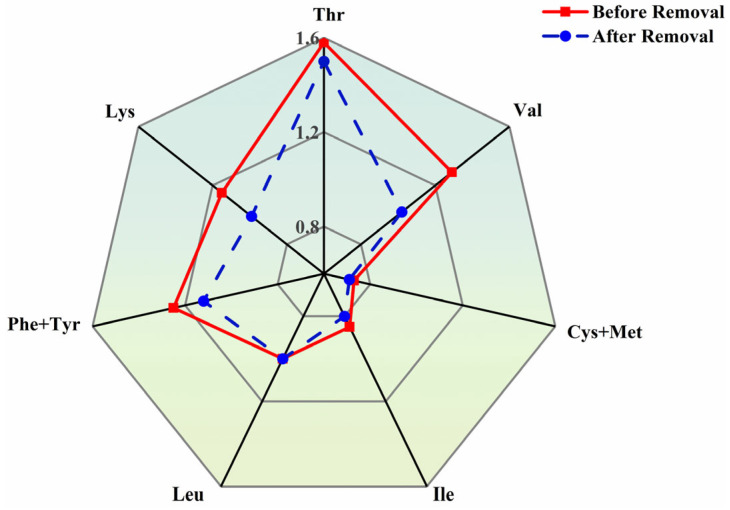
Amino acid scores of *P. haitanensis* before and after UACA treatment.

Figure 10 presents amino acid evaluation results based on FAO/WHO scoring criteria, showing that the amino acid scoring patterns of *P. haitanensis* before and after treatment were highly consistent. Cys and Met were identified as the first limiting amino acids in *P. haitanensis*, with Ile as the second limiting amino acid. The types of limiting amino acids remained unchanged after heavy metals removal, indicating that the treatment did not significantly alter the limiting amino acid characteristics of *P. haitanensis* protein, and amino acid losses were relatively balanced. Furthermore, although scores for other essential amino acids slightly decreased after treatment, the overall scoring pattern remained stable, indicating that the heavy metals removal process did not cause selective loss of specific essential amino acids, and the compositional structure remained largely intact.

Among non-essential amino acids, Glu and Asp are the most abundant in algae and contribute significantly to the characteristic umami flavor of *P. haitanensis* [67]. Furthermore, after heavy metals removal, Glu and Asp remained the two amino acids at the highest concentrations, with retention rates of 84.75% and 84.12%, respectively, indicating that UACA treatment has only a minor effect on the umami flavor of *P. haitanensis*.

Regarding nutritional components, a moderate loss of nutrients was observed in *P. haitanensis* following heavy metals removal. This observation may be explained by the fact that nutrient accumulation in algal cells largely depends on the complexation of cell walls and polysaccharides with heavy metals to form stable complexes [10]. During treatment, removal of heavy metals was accompanied by partial elution of bound proteins; additionally, UACA treatment may have caused cell wall rupture in *P. haitanensis*, leading to minor protein loss [68]. Concurrently, the contents of various amino acids also decreased, indicating that while UACA removes heavy metals, it exerts a moderate effect on protein structure and amino acid retention in *P. haitanensis*. This effect may be attributed to the presence of functional groups such as -COOH and -NH_2_ in amino acid side chains, which form chelates with heavy metals, resulting in the formation of metal–amino acid complexes. Under the combined effects of ultrasonic cavitation and CA, metal ions originally bound to amino acids may be desorbed, disrupting metal–amino acid complexes or dissolving along with metal complexes, leading to a slight reduction in amino acid content [69]. This observation is consistent with the previously reported reduction in crude protein content.

Although this process leads to some nutrient loss, its benefits are considerable from the perspective of food safety and consumer risk-benefit assessment. Heavy metals are highly bioaccumulative and non-degradable [12], and long-term exposure can cause irreversible damage to the human nervous system and kidneys, posing significant health risks. In contrast, proteins and polysaccharides are essential nutrients that are abundant and readily replaceable. Consumers can offset the nutritional loss from *P. haitanensis* by consuming other foods, such as meat and grains, in their daily diet. Therefore, from the perspective of overall dietary structure, the nutritional loss associated with this process can be mitigated through dietary diversification and does not present a substantial nutritional risk to consumers, thereby supporting the principle of prioritizing food safety.

## 4. Conclusions

This study evaluated the removal efficiencies of heavy metals (Cr, As, Cd, and Pb) from *P. haitanensis* using various physical and chemical methods and applied a combined physical-chemical approach (UACA) to reduce heavy metal content. Based on single-factor experiments, a response surface study using the Box–Behnken design was conducted to optimize the UACA process. The optimized process conditions were determined as a CA concentration of 0.11 mol/L, an ultrasonication time of 27 min, and an ultrasonic power of 270 W, achieving removal rates of 74.7% for Cr, 81.2% for As, 92.0% for Cd, and 51.2% for Pb, with residual concentrations of 0.43, 0.42, 0.058, and 0.47 mg/kg, respectively. FT-IR and subcellular structural analyses revealed that UACA removes heavy metals through the “Release–Chelation” effect mechanism, disrupting metal binding to organic matter in the cell wall and facilitating their release. SEM images showed significant alterations in the surface structure of treated *P. haitanensis*, including partial cell wall disruption, which correlated with heavy metal removal efficiency. Although protein, polysaccharide, and amino acid contents in *P. haitanensis* decreased slightly after treatment, the EAA/NEAA ratio remained above 60% and the EAA/TAA ratio around 40%, indicating that protein quality was maintained. In summary, this study provides an effective method for reducing heavy metals in *P. haitanensis* when residual levels exceed regulatory limits. The UACA process achieves synergistic removal of multiple metals, offering a practical solution to excessive heavy metal residues and providing a theoretical foundation for further research on removal mechanisms and process optimization.

## Figures and Tables

**Table 1:** Experimental independent variable factor coding and levels.

Level	Factor		
	A (mol/L)	B (min)	C (W)
−1	0.06	20	240
0	0.10	25	270
1	0.14	30	300

**Table 2:** Response surface design and results.

Num	A mol/L	B min	C W	Removal Rate (%)			
				Cr	As	Cd	Pb
1	−1	−1	0	48.17 ± 0.95	50.67 ± 1.55	70.14 ± 1.37	32.25 ± 2.08
2	1	−1	0	62.33 ± 2.22	74.46 ± 2.13	78.92 ± 1.79	37.67 ± 0.14
3	−1	1	0	65.06 ± 1.18	73.82 ± 0.45	83.33 ± 0.85	41.69 ± 1.39
4	1	1	0	69.05 ± 1.53	72.64 ± 1.84	89.34 ± 1.41	46.67 ± 0.62
5	−1	0	−1	47.29 ±1.96	61.68 ± 1.75	75.79 ± 2.41	39.98 ± 1.47
6	1	0	−1	64.75 ± 0.76	76.13 ± 0.89	86.75 ± 1.30	44.61 ± 0.25
7	−1	0	1	62.25 ± 1.81	68.72 ± 0.47	80.14 ± 0.67	41.12 ± 2.53
8	1	0	1	63.46 ± 2.12	76.13 ± 1.17	81.92 ± 1.57	45.15 ± 1.86
9	0	−1	−1	52.67 ± 0.69	63.65 ± 1.27	70.98 ± 2.25	38.13 ± 2.09
10	0	1	−1	65.25 ± 2.24	72.13 ± 0.15	83.63 ± 0.91	46.15 ± 2.41
11	0	−1	1	59.49 ± 1.64	63.65 ± 0.72	71.31 ± 0.95	36.13 ± 0.76
12	0	1	1	67.71 ± 1.16	75.69 ± 1.28	83.92 ± 1.69	47.90 ± 2.32
13	0	0	0	73.98 ± 1.96	79.54 ± 2.13	90.61 ± 1.43	49.22 ± 1.59
14	0	0	0	73.12 ± 1.24	79.69 ± 1.68	90.57 ± 1.98	49.24 ± 1.28
15	0	0	0	73.49 ± 2.16	80.06 ± 1.43	89.92 ± 1.65	49.73 ± 1.75

**Table 3:** Analysis of variance table for response surface experiment results.

Source	Cr		As		Cd		Pb	
	F	*p*	F	*p*	F	*p*	F	*p*
Model	142.79	<0.0001	284.04	<0.0001	305.81	<0.0001	115.32	<0.0001
A	221.07	<0.0001	661.24	<0.0001	361.85	<0.0001	385.00	<0.0001
B	321.08	<0.0001	603.82	<0.0001	1140.25	<0.0001	133.08	<0.0001
C	85.97	0.0002	38.13	0.0016	0.0094	0.9267	11.86	0.0184
AB	33.68	0.0021	434.47	<0.0001	7.33	0.0424	36.64	0.0018
AC	85.66	0.0002	33.64	0.0021	80.47	0.0003	7.79	0.384
BC	6.91	0.0553	8.60	0.0325	0.0015	0.9703	3.72	0.1117
A^2^	242.84	<0.0001	245.55	<0.0001	137.17	<0.0001	376.08	<0.0001
B^2^	133.62	<0.0001	468.22	<0.0001	653.79	<0.0001	92.58	0.0002
C^2^	234.21	<0.0001	172.58	<0.0001	523.26	<0.0001	38.13	0.0016
Lack of fit	6.21	0.1418	7.90	0.1144	2.32	0.3154	15.99	0.0594
R^2^	0.9961		0.9980		0.9982		0.9952	
Adj R^2^	0.9891		0.9945		0.9949		0.9866	
Adeq Pre	35.8076		58.3190		48.2786		35.7003	

**Table 4:** Subcellular distribution of heavy metals: mg/kg.

Heavy Metal		Cell Wall	Organelle	Soluble Fraction
Before Removal	Cr	0.89 ± 0.020 ^a^	0.39 ± 0.011 ^c^	0.45 ± 0.035 ^b^
	As	0.96 ± 0.027 ^a^	0.41 ± 0.019 ^c^	0.83 ± 0.038 ^b^
	Cd	0.39 ± 0.021 ^a^	0.24 ± 0.030 ^b^	0.10 ± 0.012 ^c^
	Pb	0.45 ± 0.019 ^a^	0.28 ± 0.017 ^b^	0.22 ± 0.013 ^c^
After Removal	Cr	0.11 ± 0.011 ^b^	0.20 ± 0.015 ^a^	0.12 ± 0.011 ^b^
	As	0.11 ± 0.012 ^b^	0.16 ± 0.0010 ^a^	0.15 ± 0.0090 ^a^
	Cd	0.019 ± 0.012 ^ab^	0.029 ± 0.008 ^a^	0.010 ± 0.005 ^c^
	Pb	0.16 ± 0.016 ^b^	0.19 ± 0.012 ^a^	0.12 ± 0.0020 ^c^

Different letters indicate significant differences among components (*p* < 0.05).

**Table 5:** Comparison of basic nutritional components before and after treatment.

	Crude Protein (%)	Total Polysaccharides (%)
Before Removal	27.42% ± 0.31 ^a^	26.36% ± 0.07 ^a^
After Removal	23.67% ± 0.27 ^b^	22.60% ± 0.04 ^b^

Different letters indicate significant differences in the contents of nutritional components in *P. haitanensis* before and after treatment (*p* < 0.05).

**Table 6:** Comparison of amino acid content before and after treatment.

Amino Acids	Before Removal	After Removal
Thr *	1.74 ± 0.09 ^e^	1.42 ± 0.11 ^c^
Val *	1.77 ± 0.11 ^de^	1.21 ± 0.05 ^e^
Met *	0.53 ± 0.06 ^j^	0.46 ± 0.08 ^i^
Ile *	0.93 ± 0.07 ^i^	0.76 ± 0.07 ^h^
Leu *	1.91 ± 0.09 ^d^	1.65 ± 0.18 ^v^
Phe *	1.29 ± 0.02 ^g^	0.97 ± 0.09 ^g^
Lys *	1.89 ± 0.16 ^d^	1.41 ± 0.09 ^c^
Asp	2.77 ± 0.11 ^c^	2.33 ± 0.13 ^b^
Ser	1.36 ± 0.15 ^g^	1.16 ± 0.07 ^ef^
Glu	3.41 ± 0.12 ^a^	2.89 ± 0.07 ^a^
Gly	1.69 ± 0.07 ^e^	1.37 ± 0.14 ^c^
Ala	2.93 ± 0.14 ^b^	2.26 ± 0.15 ^b^
Cys	0.17 ± 0.05 ^k^	0.13 ± 0.04 ^j^
Tyr	0.60 ± 0.15 ^j^	0.49 ± 0.07 ^i^
His	0.45 ± 0.04 ^j^	0.20 ± 0.06 ^j^
Arg	1.53 ± 0.09 ^f^	1.02 ± 0.09 ^fg^
Pro	1.11 ± 0.08 ^h^	0.77 ± 0.08 ^h^
EAA	10.05 ±0.23	7.88 ± 0.28
NEAA	16.01 ± 0.27	12.62 ± 0.37
TAA	26.07 ± 0.38	20.50 ± 0.60
EAA/NEAA	62.79%	62.46%
EAA/TAA	38.57%	38.45%

Note: * represents essential amino acids. EAA represents essential amino acids, NEAA represents non-essential amino acids, and TAA represents total amino acids. Different letters indicate significant differences in the contents of amino acids in *P. haitanensis* before and after treatment (*p* < 0.05).

## Data Availability

Due to the policies and confidentiality agreements followed by our laboratory. The raw data supporting the conclusions of this article will be made available by the authors on request.

## Supplementary Materials

The following supporting information can be downloaded at: https://www.mdpi.com/article/10.3390/toxics14050401/s1. Figure S1: Effect of rotational speed on the removal efficiency of *Pyropia haitanensis*; Figure S2: Schematic plot of the UACA removal mechanism; Figure S3: Residual analysis of the response surface model for Cr removal rate in *Pyropia haitanensis*: Figure S4: Residual analysis of the response surface model for As removal rate in *Pyropia haitanensis*: Figure S5: Residual analysis of the response surface model for Cd removal rate in *Pyropia haitanensis*: Figure S6: Residual analysis of the response surface model for Pb removal rate in *Pyropia haitanensis*. References [24,70,71,72,73,74] are cited in Supplementary Materials.

## Abbreviations

The following abbreviations are used in this manuscript:

CA	Citric acid
UACA	Ultrasound-assisted citric acid
EDTA	Ethylenediaminetetraacetic acid
HAc	Acetic acid
LA	Lactic acid
