# Supplementary material for: Release–Chelation Effect of Ultrasound-Assisted Citric Acid Treatment on Heavy Metal Removal Efficiency in Pyropia haitanensis
Source: Toxics. 2026 May 7;14(5):401. doi: 10.3390/toxics14050401 (PMC13211275; doi:10.3390/toxics14050401)
Supplement: Supplementary file 1 [file toxics-14-00401-s001.zip › toxics-4218930-supplementary.pdf]

3.2.2. Mechanical stirring

Agitation washing represents one of the most widely employed physical cleaning techniques in the food processing industry. By exploiting the rotational motion of the agitator, this method ensures intimate contact between the food material and the cleaning solution, thereby facilitating the removal of surface sediments and suspended particles to achieve an effective cleaning outcome. In the present study, a simulated agitation washing apparatus was developed, and experiments were conducted across a range of rotational speeds and processing durations to investigate the effects of agitation speed and processing time on the removal efficiencies of different heavy metals. The results are presented in Figure S1. The results demonstrated that the removal efficiencies of Cr, Cd, and Pb increased with increasing stirring speeds. Fouda-Mbanga [70] previously reported that heavy metal removal efficiencies increase with higher initial concentrations, temperatures, adsorbent dosages, contact times, and stirring speeds, consistent with the present findings. Conversely, for As, the removal efficiency gradually decreased as the stirring speed increased within the 300-500 rpm range. Lan [24] reported that there exists a threshold stirring speed for the efficient removal of copper (Cu). This phenomenon may be associated with the chemical forms of As present in algae. Yu [71] reported that As in seaweed exists in both inorganic and organic forms, including arsenosaccharides. According to Park [72], inorganic arsenic is readily soluble in water following boiling and saltwater soaking, whereas organic arsenic is more resistant to extraction. Integrating these findings, it can be inferred that inorganic arsenic is more readily released from algal cells into the solution, whereas organic arsenic remains more stable and less amenable to removal. Therefore, under stirring conditions of 100-300 rpm, inorganic arsenic may be efficiently released from the cell surface into the solution. However, at stirring speeds of 300-500 rpm, excessive agitation may damage the cellular structure of *Pyropia haitanensis*, potentially leading to the re-adsorption of released inorganic arsenic onto the cell surface through passive adsorption [73].

Consequently, excessively high stirring speeds may compromise the effectiveness of

subsequent arsenic removal. In summary, under conditions of 30 min at 400 rpm, the removal efficiencies of the investigated heavy metals were optimal, with residual concentrations of Cr, As, Cd, and Pb measured at 1.33 mg/kg, 1.79 mg/kg, 0.54 mg/kg, and 0.76 mg/kg, respectively[74].

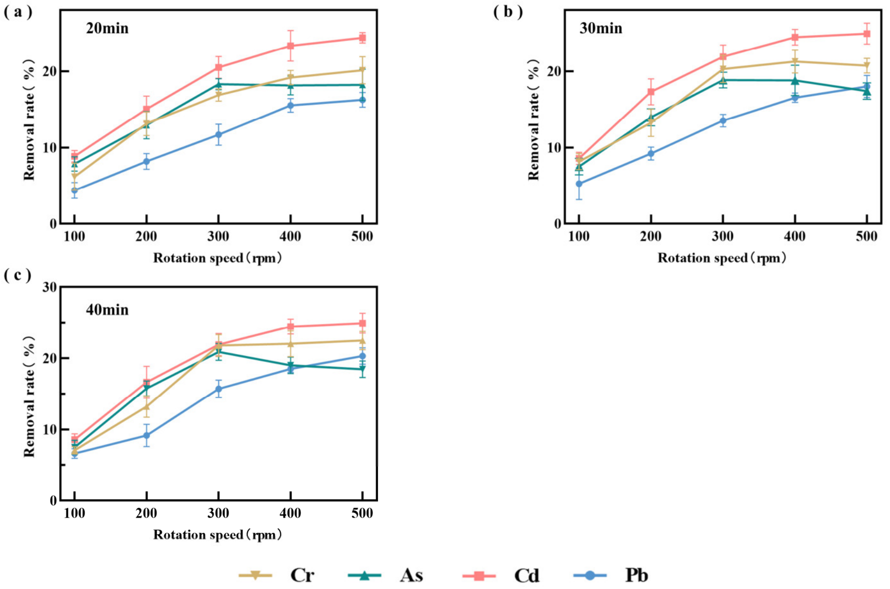

Figure S1: Effect of rotational speed on the removal efficiency of *Pyropia haitanensis*

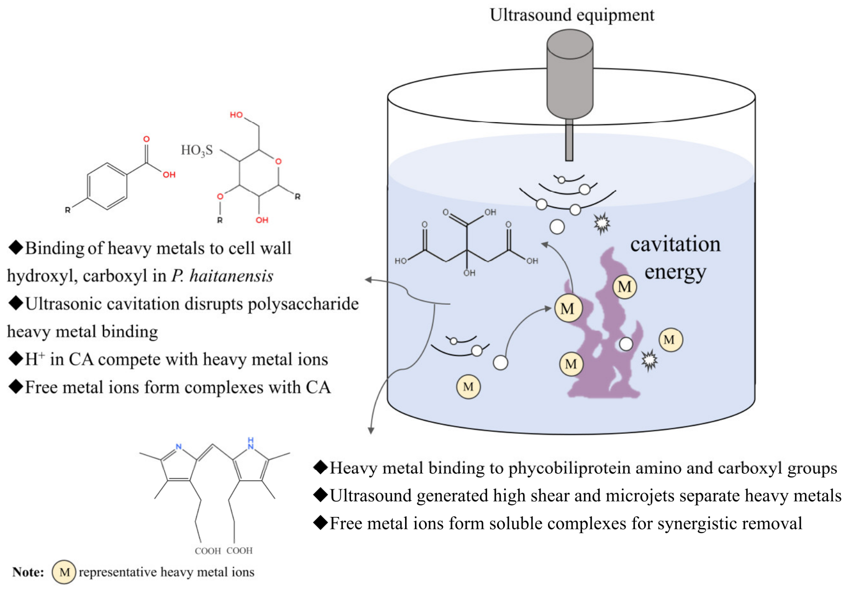

Figure S2: Schematic plot of the UACA removal mechanism

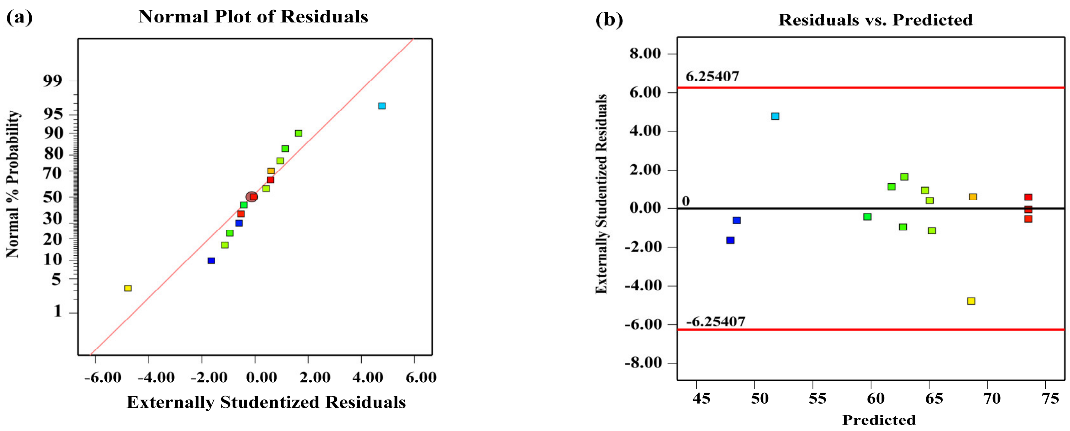

Figure S3:. Residual analysis of the response surface model for Cr removal rate in *Pyropia haitanensis*: (**a**) Normal probability plot of residuals, indicating that the residuals approximately follow a normal distribution; (**b**) Residuals versus predicted values plot, showing no obvious patterns, confirming homoscedasticity.

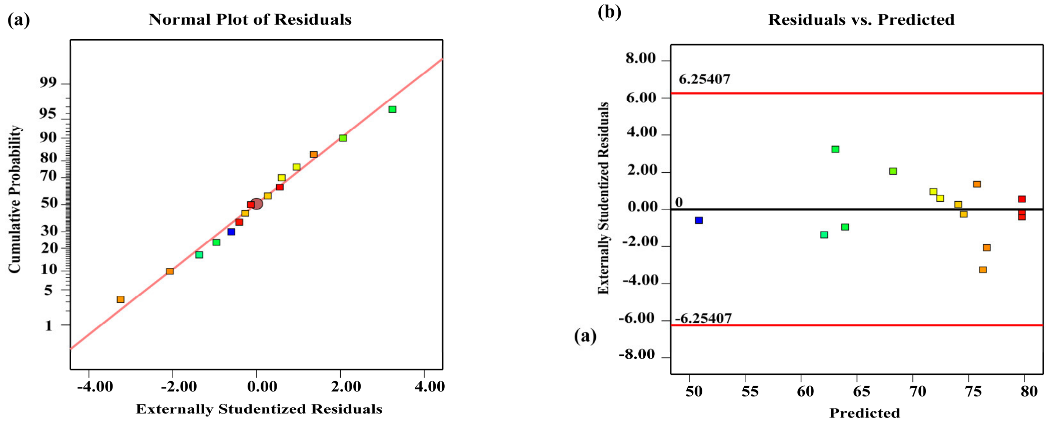

Figure S4: Residual analysis of the response surface model for As removal rate in *Pyropia haitanensis*: (**a**) Normal probability plot of residuals, indicating that the residuals approximately follow a normal distribution; (**b**) Residuals versus predicted values plot, showing no obvious patterns, confirming homoscedasticity.

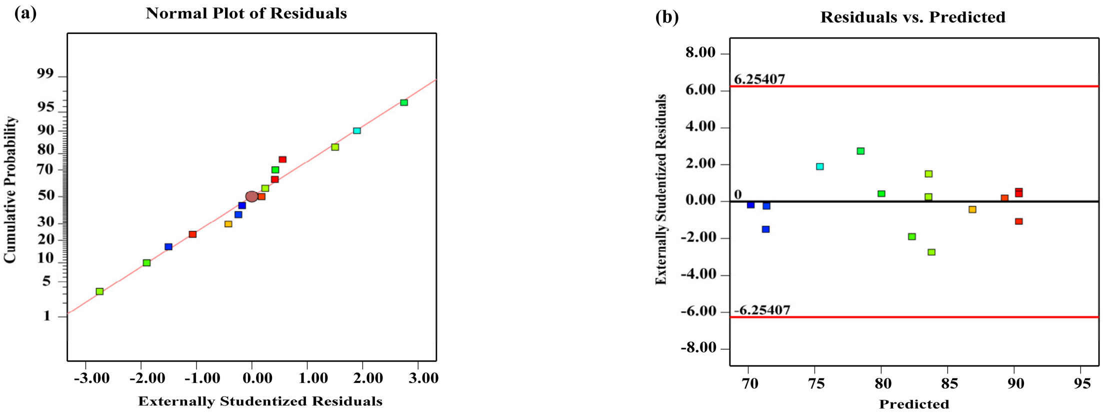

Figure S5: Residual analysis of the response surface model for Cd removal rate in *Pyropia haitanensis*: (**a**) Normal probability plot of residuals, indicating that the residuals approximately follow a normal distribution; (**b**) Residuals versus predicted values plot, showing no obvious patterns, confirming homoscedasticity.

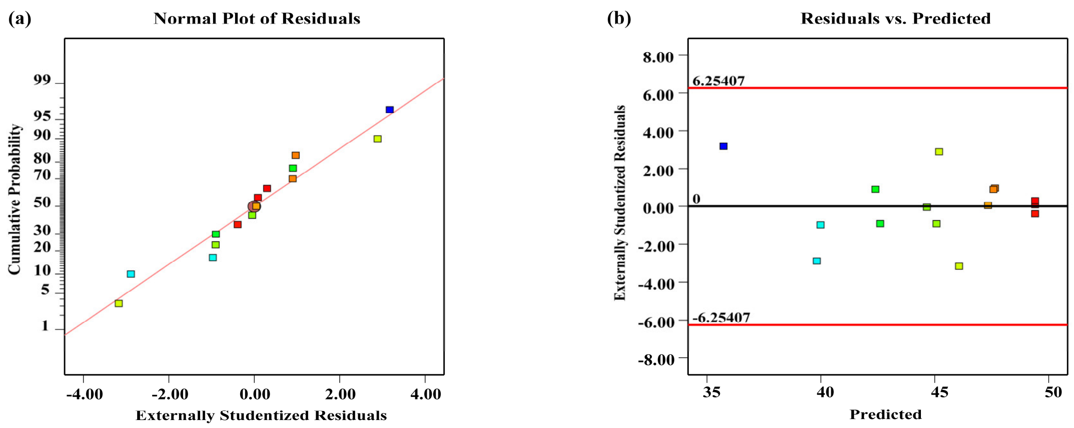

Figure S6: Residual analysis of the response surface model for Pb removal rate in *Pyropia haitanensis*: (**a**) Normal probability plot of residuals, indicating that the residuals approximately follow a normal distribution; (**b**) Residuals versus predicted values plot, showing no obvious patterns, confirming homoscedasticity.

References

[24] L. Xiaobing, C. Jun, X. Yang, H. Fenglong, C. Changzhong, L. Dongdong, J. Jianhong, D. Bin, Investigation on the Removal Performances of Heavy Metal Copper (II) Ions from Aqueous Solutions Using Hydrate-Based Method, Molecules 28(2) (2023) 469-469.

[70] B.G.F. Mbanga, T. Velempini, K. Pillay, Z.T. Ngeva, Heavy metals removals from wastewater and reuse of the metal loaded adsorbents in various applications: A review, Hybrid Advances 6 (2024) 100193-.

[71] Y. Yu, A.M. Rodriguez, G. Zhou, D. Barrón, À. Sahuquillo, J.F.L. Sánchez, Survey of arsenic content in edible seaweeds and their health risk assessment, Food and chemical toxicology : an international journal published for the British Industrial Biological Research Association 187 (2024) 114603-114603.

[72] P. Ga-Young, K. Da-Eun, D. Munkhtugs, S. Choonshik, K. Gil-Jin, C. Myung-Sub, Reduction of total, organic, and inorganic arsenic content in Hizikia fusiforme (Hijiki), Food science and biotechnology 28(2) (2019) 615-622.

[73] C. Zhonghao, O.A. I., R.D. W., O. WenDa, Y. PowSeng, Remediation of Heavy Metals in Polluted Water by Immobilized Algae: Current Applications and Future Perspectives, Sustainability 15(6) (2023) 5128-5128.

[74] Z. Wenhao, S. Frederick, Z. Cunshan, Use of Ultrasonic Cleaning Technology in the Whole Process of Fruit and Vegetable Processing, Foods 11(18) (2022) 2874-2874.
